# The Long Fox Lecture: Dermatitis from without and Dermatitis from Within

**Published:** 1906-12

**Authors:** Alfred J. Harrison

**Affiliations:** Consulting Physician, late Physician to the Dermatological Department, Bristol General Hospital


### THE LONG FOX LECTURE:

THE THIRD ANNUAL LECTURE ARRANGED BY THE COMMITTEE OF THE LONG FOX MEMORIAL,

DELIVERED IN THE MEDICAL LIBRARY, UNIVERSITY COLLEGE, BRISTOL, ON DECEMBER 6TH, 1906.

F. RICHARDSON CROSS, M.B. Lond., F.R.C.S., *in the Chair.*

BY

ALFRED J. HARRISON, M.B. (Lond.), *Consulting Physician, late Physician to the Dermatological Department, Bristol General Hospital,*

ON

### DERMATITIS FROM WITHOUT AND DERMATITIS FROM WITHIN.

IT is with feelings of great distrust in my competence, but with deep regard to the honour that has been put upon me, that I venture to come before you to-day. The trust—if I may so style it—which has established this "Long Fox Memorial Lecture" has taken upon it to arrange for an annual address, the subject of which shall have a very marked professional bearing, and which, as far as it practically can, shall enhance the good of the profession by dealing with some subject of more or less utility to the hard-worked members of it, and amongst these the general practitioners must be very prominent, for whatever helps them must do honour to the profession at large, and we shall best perpetuate the memory of our deceased friend by keeping these important objects in view.

Edward Long Fox was a man of large heart and general philanthropy—all who knew him must feel this—and personally, I can speak as one who has been the recipient of much kindness and hospitality from him, and has often been present also at those large and interesting gatherings of his professional brothers which he loved to assemble. Never was he seen to better advantage than when surrounded by such an assembly, for his heart's best sympathies were there. How often do we say and feel with our late Poet Laureate—

> "Oh! for the touch of a vanish'd hand
> And the sound of a voice that is still."

But if that voice is still, the spirit that animated that voice can never be still. It is with us now, and will be ever with us when we are endeavouring—feeble though the effort be—to advance the interests and knowledge of that profession which he loved and adorned.

The subject which I have chosen is one which is closely connected with what I may term, my more especial department of medicine—dermatology, and which has interested me very much during recent years. It can be best summarised in the terms, "Dermatitis from without and dermatitis from within," or, in other words, irritation or inflammation of the skin caused by bodies applied to or in close proximity to the surface of the skin, and the effects produced on the skin by irritants taken within the system, either by the stomach or by the lungs, or injected into the deeper tissues of the skin.

Now this is a very large and comprehensive subject, for in its full bearings it would seem to embrace all the exanthemata, the erythemata, erysipelas, most eczemas, some new growths, lupus, tuberculosis, leprosy, elephantiasis, the action of toxines, and parasitic affections, whether produced by vegetable or animal parasites. All these cannot be dealt with or even touched upon, for if they were the time allotted for this address would be very insufficient, and so far as I am concerned, would leave behind a lasting memorial of stuffiness and confusion, to say nothing of exasperating your patience beyond all bounds. I shall therefore limit my remarks to bringing before you a few cases which I have come across in my own experience, or that medical friends have had in their career of work and which I may have seen in conjunction with them, or they have kindly permitted me to use to exemplify my subject.

I do not wish to burden you with a description of the structure and physiology of the skin, but I want to draw your attention to one view. We know the skin is a very important and extensive covering of this complex body of ours, but it is also the parade ground for a large number of complaints. Think how many things we recognise or attempt so to do by their behaviour or manifestations here, and it can therefore be no wonder that with the best and most experienced of us doubts and confusions should often arise. If we look at a body of infantry or cavalry parading on their exercise ground they appear to us wonderfully alike at first glance, and yet every one of these individuals has a separate identity and very marked personal differences, and the same arguments apply to the appearances of various affections on their parade ground—the skin. Let me very briefly remind you that this said skin has its own peculiar structure, its own nerve endings and vascular supply, and that it is complicated by an enormous number of glands and hairs, each and every part fulfilling its own special mission, and yet being implicated in derangements and diseased processes, whether invaded, in the first instance, from within or from without, or in both ways. It is said that a good shepherd will recognise each individual sheep in a large flock. Let it be our province as shepherds of more valuable sheep to endeavour to name every pathological process which may display itself, however confusedly, upon the human skin. This accomplished, many things which are now obscure will become as clear as daylight.

The surface of our skin is not very irritable, it will stand a fair amount of friction in health without any serious resentment; but in certain individuals, coupled with some peculiarity or idiosyncracy, temporary or more lasting, a very little irritation will be followed by very marked symptoms. A patient will come before you complaining that the face flushes very much if it be rubbed only moderately after washing or bathing, that an uncomfortable redness will come on and continue with more or less persistence, or that a slightly over-heated room will produce similar symptoms.

Many years ago a young fellow, in the early teens, E.G., quite healthy-looking, came to me at the Bristol General Hospital, complaining chiefly of these uncomfortable symptoms. He was an active young fellow, very fond of games and swimming, but if he did either his face and the skin of his body would become very red and hot, and then his companions would laugh and poke innocent fun at him. Could I do something for him? He had a younger brother who suffered much in the same way. Their general health seemed good, and there was nothing unhealthy or particularly trying connected with their occupations. I tested the reaction of his skin, and I found out that if I traced out his initials, E.G., on his back over the shoulder-blades with the blunt end of a penholder, the following manifestations took place. Very shortly a diffused redness would appear, the surface of it would next fade away and contract, leaving the letters as marked red lines, but very slightly raised. Special blood vessels had been irritated and became congested. The congestion increased, and red wheals formed. A little later the redness began to pale, but was succeeded by very marked white wheals. The red globules of the blood had passed on, but serum had been poured out, and gradually the white wheals became very evident to the eye, and so discretely prominent that a blind man would have no difficulty in deciphering the letters by touch. In about a quarter of an hour or longer the whole thing would have faded away, and "left not a wrack behind." From time to time I have examined a large number of these cases, men and women, who complain of flushing and burning of the face when they go into hot rooms or drink hot liquids, all subjects of this condition, to which the following terms have been applied: "Dermographia," "Auto-graphia," and "Factitious Urticaria"; and from my experience I should be inclined to lay down the following rule: That the slower the congestive process, the more marked are the wheals, and the more persistent. When there are no symptoms of disturbed health, treatment of these cases is not very satisfactory; and with regard to these brothers, I did not succeed in doing much, but later on they got into the Navy, and in a short time they got rid of their skin trouble; and when last I heard of them they were fine, manly young fellows, excellent types of our best-looking and healthiest sailors. I used the term "idiosyncracy" advisedly just now, and I have occupied some little time in details because this peculiarity is one of the simplest forms of irritation of the skin, much simpler than that produced by the common stinging nettle on most skins. We can hardly term it "dermatitis," but it and some kindred conditions, will be followed by dermatitis, caused by the rubbing or scratching of the skin, which many individuals seem unable to restrain. You might be inclined to assume that a skin so prone to be irritable would respond to many other irritants. This by no means follows as a rule; such persons are not especial victims of flea, bug, or mosquito bite, nor of irritant foods or drugs taken into the system. There is another lesson to be inculcated. Supposing a boy with this "dermo-graphia" received a caning—and it need not be a severe one—from his master, and the boy went home shortly afterwards. Picture an impetuous father or an over-anxious mother examining the child. What indignation and feelings of resentment would be stirred up. I well remember the late Sir Wm. Gull relating such a case, and those who knew him may remember how dogmatic he could be. He was concerned where a schoolmaster was accused of having unduly and cruelly chastised a boy. Bringing his knowledge and acumen to bear, Dr. Gull showed very graphically that the boy was the subject of dermo-graphia. Hence, you see, there is a medico-legal side to the condition.

Take another case, which you will shortly see advances a little this state of irritable skin. Miss F. had very marked irritation of the hands, arms, neck, and face, which made her life—a very active one—rather an uncomfortable one. Autographia was very marked, She could initial her name on her arms in a very palpable manner. There was some disturbance of the digestion, but really very few morbid symptoms. She improved slowly, but uncertainly. She would vary without any obvious cause. In the end we discovered that what kept up the irritation, but was certainly not the initial or sole cause, was the presence in a conservatory, where she was very fond of going and of looking after certain favourite plants, of the Primula obconica. A change from home, and thus complete absence of the obnoxious plant, soon re-established her usual healthy condition. Some persons are affected by one thing and some by another, as the long list of irritants on the diagram before you must indicate, and these and similar conditions go a long way to explain those individual peculiarities where various foods and drinks, and drugs and plants, etc., cause very peculiar and very often alarming symptoms. The proverb, "What is one man's meat is another man's poison," is often strikingly illustrated. The poet Cowper said—

> "Skins may differ, but affection
> Dwells in black and white the same."

But he was not a dermatologist, otherwise he must have said—

> Skins do differ, and affections
> Differ too, by no means
> In the black and white the same.

Some years ago I was asked to see in consultation a builder's wife who had suffered for several months, off and on, from the following symptoms. She lived at Westbury, in a new house which her husband had recently built for their own occupation. Apparently whilst in fair health Mrs. M. would get great irritation of the hands, face and neck; they would become very red, and the face and eyes very œdematous, and so much so that the condition was regarded as of an erysipelatous character, and especially as there was sometimes a distinct rise of temperature and even restless nights. Under treatment and rest she would improve, and then apparently without any cause these same symptoms would recur. These relapses went on for some months. Drains were suspected, but there seemed no fault with them; and no article of food or drink could be singled out as the cause. On one occasion shortly before I saw her this took place: she had had an attack, and was kept up in her bedroom for several days as a wise precaution, and was apparently well again. In the midst of all these sanitary surroundings, where everything had been carefully done to promote and continue well-being, another severe attack came on. Congratulation was succeeded by dismay and despair. What could be at the bottom of this dark tribulation? When I heard the history of the patient, and noticed her symptoms, I thought to myself, "Surely this irritable condition of hands and face is brought on by some plant or drug," and the Primula obconica seemed to loom out of the obscurity. I saw no such flowers about her room. Had she a greenhouse? Oh, yes! and she was very fond of going there, up in the garden, when she was able to do so. We went to the greenhouse, and there were many pots of the objectionable primula. But what about the attack in the bedroom? A dear old nurse looked after her, and one day said to her, "Why, mum, as you are not allowed to go out to your greenhouse, and you do love them flowers, I 'll bring them to you." She did, and amongst them some of the Primula obconica. The prescribing of a little soothing lotion, combined with total banishment of the exciting cause, effected a complete cure, and the laying of all apparitions of dismay and despair. I saw her medical attendant quite recently, and he assured me she had had no recurrence of the attack. Recurrence, more or less, will happen after some irritants, even when the exciting cause seems absent, but this point shall be brought out by and by.

On October 20th this year an exceedingly instructive case was related by Dr. Pope, of Winnipeg, in which a lady of 64 had several recurring attacks of what was supposed to be very severe eczema, but which were really caused by Primula obconica! I could relate many more cases of dermatitis caused by this plant, but I must not weary you, and they would throw no more additional light on the subject. They are not very unfrequent, and every now and then the busy practitioner may come across a case: he is the man to do so. He needs eyes everywhere, and a mind as alive as possible to meet all kinds of subtle attacks! Some years ago a would-be critic—non-medical, of course, but with a sense of knowledge far beyond the requirements of the average medical man—wrote a book (it came out about the time of a small-pox scare) to show the uselessness of vaccination and revaccination. His statistics seemed plausible and his arguments at least specious. He had drawn his conclusions without making allowance for different ratios of population. For example, A was a town renowned for its careful vaccination, whilst B was notoriously careless, and yet A had as many cases of small-pox as B. So it might have, so far as merely numbers of cases were concerned; but if the population of A was ten times that of B, there ought to have been at least ten times the number of cases in A. We are not going to pursue this question more than to say that he termed vaccination and revaccination the "golden calf" of Harley Street. I should say, if any plant deserved the simile, Primula obconica was the golden calf, or gold-laying hen, of dermatologists!

The long list of plants which are capable of causing "dermatitis" shows that they are many in number, and as more exact knowledge goes on the number must be increased. At least two species of "Rhus," or "sumach," belong to this category—the "venenata" and "toxicodendron." In America, where the plants and trees grow largely, cases of poisoning are not unfrequent, but in this country they are very much rarer. The following is a very interesting case, and for which I am indebted to Mr. Arthur Prichard, who read a paper on the subject of poisoning by Rhus venenata before our Medico-Chirurgical Society some fifteen years ago.[1] The victim was a curate at Bradford-on-Avon, aged 25. His study was much overshadowed by a fine specimen of the tree, which grew in the vicarage garden. Hearing that a gardener, three years before, had suffered from blood-poisoning after cutting off a limb from the same tree, he started on the work with a certain amount of misgiving, and with a good deal of caution, working at the tree for a short time every day for a week. The old gardener helped him the first day, but he fell ill with severe dermatitis, and was bad for a week. At the end of a week the curate got the tree down. He noticed that the sap, which was very profuse, made his hands black (many of the sumachs are used for tanning and dyeing purposes), and he subsequently wore gloves. One of the days some of the juice touched his face, and he wiped it off with his gloved hand. The next day he had three lines of redness on his cheek, which became worse and worse for several days, producing very much the appearance of erysipelas of the face. A few days later he was so ill that he had to keep his bed. The arms, genitals, and thighs were attacked—first by a red rash, then bullæ appeared,

[1] *Bristol M.-Chir. J.*, 1891, ix. 22.

suppurated and burst, and large crusts formed, the exudation from under them being very profuse. Fortunately he was able to take nourishment well, and his strength kept up. His temperature was never 103°, nor did his pulse exceed 112. He was not convalescent for more than a month, and there is no doubt his life was in peril at one time. Dr. J. C. White, of Boston, America, has collected notes of many cases, and amongst them the case of a young lady who got a recurrence in the spring from wearing the same dress she had worn during an attack in the summer. Arnica had a great repute some years ago, and was said to be almost a specific for healing and dispersing bad contusions and sprains. Like many other vaunted remedies its reputation soon vanished, and several hospital surgeons and physicians tested very carefully its merits or demerits, and amongst them was the late Sir Alfred B. Garrod, who showed that any good there was in its application arose from the spirits of wine which was used in making the tincture; nay, more, that a lotion with the amount of spirit was often more efficacious than when combined with arnica; and there was sound reason in this, as the following case—and there are many such-like—surely shows.

Mrs. W., aged about 58, very healthy and robust, last July, sprained her left ankle very badly by slipping down some stairs. She had a great deal of vitality and brain energy, and like many such persons, disliked quietness and confinement, so she began to get about as soon as the very painful swelling began to subside, and no great harm resulted except that the ankle gave her much discomfort at times. To alleviate matters—for the intention was good—a would-be-wise lady friend recommended her to apply some tincture of arnica to the part. This she did, and being a lady of strong measures, she "slopped" (the expression is hers) a quantity of the so-called remedy over the parts several times, and then one very hot Sunday walked twice to a church—a good distance—to hear a favourite preacher. The next day the ankle and parts around became swollen and painful, dermatitis was set up, and spread all up the leg, on to the body, the chest, neck and face, and the arm, involving the whole of these parts, and also invading the right half of the body, but in a much less degree. This was about the middle of September, and it was then that I saw her. Her face was red and swollen, the neck and chest, and in a less degree, the back, was covered with a red rash which, from its appearance, might very easily have been mistaken for that of scarlet fever. The humeral portion of the left arm was affected, and the right arm slightly, but the *pièce de résistance* was the left foot and leg, which were greatly swollen and sodden, the surface of the skin being broken down here and there and discharging a viscid fluid. She was hot, restless and feverish, and highly dissatisfied with the treatment she had been induced to try. Possibly what aggravated her condition was a gouty inheritance, for before she quite recovered she suffered from gouty manifestations. Soothing lotions and rest in bed soon began to relieve her, but several weeks elapsed before she could be pronounced well, and I do not think she is likely to believe in the soothing virtues of arnica again!

Then there is another plant much more familiar to us than arnica, which in many of its species is very well known to us in this part of the country, for the ampelopsis lives in this part of England, and gives a glorious radiance of colour to a great many of our houses, from a pleasant greenness in spring-time, a darker luxurious greenness as summer advances, to bidding us farewell in the autumn, in all the hues of russet red and brilliant vermilion. Well, every now and then you come across cases where — and especially in the autumn, when probably gardeners handle the leaves more—great irritation is set up on the hands and fingers, and then, by contact of the hands upon the face and neck also. I have come across many of these cases both in this locality and in Kent, and I expect they are not unfrequent where the genus flourishes, and the one most concerned seems to be the A. quinquefolia, or Virginian creeper. These plants should be borne in mind when dealing with cases of so-named obscure eczema. They are manifestations of true plant "dermatitis."

Only this summer I take to myself the credit of having laid another "apparition" of irritation, but then I had a very intelligent medium to work with. I was attending a patient at Westbury-on-Trym last summer, Mr. S., in consultation with his medical man, for an attack of pityriasis rubra, which complaint I shall have to refer to shortly, and one day when he was convalescing he told me that nearly every year, in the late summer or early autumn, he got a curious irritation on his hands, the right more than the left, and he suggested that it might be caused by some plant in his garden, because he was very fond of working about amongst his conservatories and flower-beds. Everyone who knows Mr. S. must know what an enthusiastic and intelligent gardener he is. I mentioned several likely plants which might be the exciting cause, but he thought it could not be any one of these. I said, "Well, you are an observant man, and between us we ought to find out the enemy!" I saw him a few weeks ago, and by careful observation and elimination he had found out the culprit. He had then the remains of an irritation on his right thumb and slightly on the first and second fingers, which had been much worse. "The skin had been killed," he said, and no formula of words could express the condition better. He took me into his garden and showed me many specimens of a calendula—Tagete's marigolds, and especially one—the Legion of Honour. It is an erect bushy plant, six to eight inches in height, a very free flowerer, ornamental, and rather strong smelling. Some properties in it acted as an irritant to his skin, and the reason why the right hand was affected more than the left was because he was in the habit of pulling off the dead or drooping flowers with this hand. So this plant may very well and reasonably be added to the list of irritants.

Then there are many people who cannot handle tomatoes and chrysanthemums without getting irritation of their hands and secondarily of other parts. I saw a very intelligent old gardener, who is a victim to chrysanthemum rash. Whenever he handles them he gets redness, swelling and œdema of his hands and face. The Atropa belladonna is another irritant to some persons, but more especially is this the case when a liniment or ointment of belladonna is rubbed on the skin, or when the drug in some of its various forms is taken internally. The rash usually produced is an erythematous one, very much simulating the rash of scarlet fever, and in the early days of that curious doctrine, *Similia similibus curentur*, belladonna was to be the prophylactic against scarlet fever. However, that bubble was soon burst. Had it not, what a rivalry there would be in these days, when there is such a long list of drugs and toxines which are capable of producing very good imitations of scarlet fever rash!

The oil of copaiba is a well-known rash producer, but I do not wish to dwell much upon it except to record this, that at the hospital, many years ago, I saw an instance of this rash—I think the most marked I have ever seen—in a girl who worked at some capsule manufactory in Bedminster. She was a fair-haired, fair-skinned girl, very healthy and with a very enviable condition of skin; but the amount of rash she displayed over arms, body, face and legs was very remarkable—blotches and wheals. She assured me, and I had no reason to doubt her word, that she had never taken copaiba. I think she gave up this work and got employment in other processes. Very much akin to this is the case of a girl, aged about 18, whom I admitted into the hospital. She had a lovely white skin, but it had become deformed by an extensive erythematous rash caused by use of some of the lower grades of petroleum. She was employed at some well-known rope works in Bristol, and only quite recently had this substance been used. She was in the hospital some time because she got several relapses, apparently without any renewing cause. We tried to exclude all possible fallacies, but I have known this occur on several occasions when all exciting causes could apparently be excluded.

I am sure you will expect me to say something about "iodide" and "bromide" rashes, because these happen not unfrequently, and in their more advanced forms present some of the most severe symptoms of "dermatitis" known, when we have excluded virulent small-pox, *i.e.* small-pox unmitigated by the efficacy of vaccination. In these drug manifestations you may have great disturbance of skin tissues

### Table A.

### LIST OF DRUGS, APPLIED EXTERNALLY TO THE SKIN OR TAKEN INTERNALLY, WHICH MAY CAUSE DERMATITIS.

---

Acids (Benzoic, Boracic, Carbolic, Nitric, Phosphoric, Tannic).

Alkalis (Potash, Soda, Ammonia, Lithia).

Anilin (Dyes).

Antifebrin.

Antipyrin.

Arnica.

Arsenic.

Atropine.

Belladonna.

Bichromate of Potash.

Borax.

Bromides.

Cannabis indica.

Cantharides.

Chinolin.

Chloral.

Chloralamide.

Chlorate of Potash.

Chloroform.

Chrysarobin.

Condurango.

Copaiba.

Croton oil.

Cubebs.

Digitalis.

Ergot.

Guaiacum.

Guarana.

Hydrochlorate of Paraphenylene diamene.

Iodides.

Iodoform.

Jaborandi and Pilocarpine.

Lacto-phenine.

Mercury.

Mustard.

Opium and its Alkaloids.

Orthoform.

Petroleum.

Phenacetin.

Phosphorus.

Quinine and its Alkaloids.

Salicylic Acid and Salicylates.

Salipyrin.

Santonin.

Savin.

Stramonium.

Strychnia.

Sulphonal.

Tar, Terebene, Turpentine.

Tartar Emetic.

---

Certain Toxins and Antitoxins.

### Table B.

### LIST OF PLANTS, &c., WHICH MAY CAUSE DERMATITIS.

---

*A*. Ampelopsis, especially Quinquefolia (Virginia Creeper).
Chrysanthemums.
Cocos-wood (Flute-makers).
Cypripedium angelica.
Heracleum giganteum.
Hyacinthus orientalis.
Primula obconica.
Rhus toxicodendron (Poison Ivy or Oak).
Rhus venenata (Poison Elder or Dog-wood).
Roses (especially in America).
Tagétes (especially "Legion of Honour").
Urticæ.

*B*. Stings of Bees, Wasps, Hornets.

*C*. Bites of Tarantula, Fleas, Bugs, Mosquitoes, &c.

*D*. Hairs of Caterpillars.

*E*. Animal and Vegetable Parasites.

*F*. Secretions from Physalia, Jelly-fish, &c.

and swellings and tumours even to a very disfiguring degree. The outbreak may be spotted, papular, vesicular, pustular, petechial or bullous, or a combination of several of these forms, and even sub-cutaneous abscesses may show themselves. They are chiefly developed upon face, neck, trunk and arms, but no part of the whole surface is exempt, and it is in scar tissue following wounds that rashes first show themselves in a very marked way; the case may even end fatally.

Silver salts, especially the nitrate, when taken for some long time will affect the skin, but the "dermatitis" strictly speaking must be slight, and these cases are not very likely to be seen now, as other drugs have superseded silver nitrate, and if silver salts are given or used externally, such as argentol or protargol and some others, these and the solutions are said not to be precipitated by NaCl, or to cause coagulation of albumin.

Chrysarobin is another drug which I can speak about as a skin irritant. It is used very advantageously in many cases of obstinate psoriasis, and in one case I remember, an enthusiastic patient, notwithstanding warnings, employed it too vigorously, with the result that she was the best-marked example of erythema of the thigh, abdomen and chest I have ever seen. Before leaving this part of the subject there is one observation, inference or deduction, may I call it, I should like to make. There is not unfrequently considerable discussion about dieting in eczema, or so-called eczema. I do not wish to deride or diminish the importance of this in many cases, but I have seen, and I am sure a great many of you have seen, people who have been precluded from taking this or the other thing in their dietary, cut off from sugar, certain meats, certain fish, alcohol, tea, coffee, fruit, etc., until life seems to be scarcely worth the living; but with it all the complaint seems to get rather worse than better, and they themselves get weaker and weaker, and more and more miserable. Might not some of these cases come under the head of some subtle existing and often persisting irritation corresponding with some of the drug or plant or toxines we have been regarding? Eczema is truly "dermatitis," but "dermatitis" may not be eczema!

Many years ago, soon after I was qualified, I went out as ship's surgeon to Tasmania and back, and amongst many things we collected at sea—a sailing vessel gives you so many more opportunities than a rapid, rushing steamer—were several specimens of the "Physalia utriculus," or Portuguese man-of-war. They are not uncommonly seen in fine weather in warmish waters. They are composed of a large, float-like body, beautifully tinted—a kind of swimming bladder—and beneath this are a number of tentacles. Well, one day we had collected several of these interesting creatures, and we put them into a child's bath, and kept them in it for some time, and then tossed them out to sea again. A young infant was washed in the bath that evening, and very soon afterwards was tormented with severe nettle rash, or urticaria. No doubt the bath had not been thoroughly cleansed, and some excretions from the "Physalia" must have been the cause. You know that many jellyfish are capable of doing the same thing. Well, this brings me to make a few remarks about caterpillars. Many species are capable of setting up much irritation of the fingers in children who handle them, and this summer I came across an amusing description of such cases. I am quoting from the *British Journal of Children's Diseases* for last July :—

"Wednesday, June 20th, was a red-letter day for the Chelmsford Rural Council and their medical officer, for the news had been flashed abroad on the wings of the mode of motion, known as electricity, that a new disease had been brought to light in Essex. It is permissible to employ the metaphor of wings, for this wonderfully novel complaint, called 'Caterpillar's Rash,' had been traced to caterpillars. . . . There is a little mystery about the Essex disease which must first be cleared up. In the published paragraph it is referred to as caterpillar's disease, but in the account of it the public is gravely informed that 'twenty children were infected with it,' which statement makes things still more complex and the brain to stagger. If caterpillars in Essex have got a rash, then it is high time the matter should be seen to and dealt with sternly but firmly. A society for the prevention of rashes among caterpillars should be at once started, and money collected, say, from the butterflies of fashion. Or some budding Pasteur from the Lister Institute should immediately take a parliamentary ticket (return) to the locality in Essex suffering from this plague and investigate this new silkworm disease. On the other hand, the caterpillars may be enjoying their normal health, and not affected by 'Dermatitis larvæ Chelmsfordii' at all, and the children turn out to be the victims of the complaint. Now to business. Over fifty years ago an industrious German investigator, Will by name, analysed the delicate hairs of various kinds of caterpillars, and stated that he had found formic acid in them, which he asserted was at the bottom of the mischief. Since then many others have worked at the subject, and contended that the irritating substance found in the hairs and in the glands situated at their roots was allied to cantharidine. . . . It will interest the Chelmsford Rural Council to know that the caterpillar of Cossus ligniperda can elegantly expectorate a caustic greenish sputum of pungent odour to a distance of two feet, As to the watery eyes mentioned in the Essex epidemic. Meixner has written a dissertation on caterpillar ophthalmia. Further, the caterpillar of a common or garden butterfly, Pieris brassicæ, has given rise to colic, salivation, stomatitis and other alimentary symptoms when swallowed by cows and horses. According to Livingstone, the Bushmen employed a small caterpillar to poison their arrows with. Again, the silkworm caterpillar excretes an irritating substance well known to the workers in silk factories."

These rashes produced by caterpillars, either directly or indirectly, are very closely allied to those manifestations which are caused by the antitoxin of diphtheria and toxins used or developed in what is termed serum-therapy. In carrying out these treatments rashes are often developed upon the skin, notably by the antitoxin of diphtheria and by injections into the skin of tuberculin. The sub-committee appointed by the Clinical Society made a short time ago a very interesting report on the rash and some other effects produced by the antitoxin of diphtheria. Out of 633 cases reported upon, an erythematous or urticarial rash, in some cases followed by petechiæ, came on in 220 cases, or in 34.7 per cent. In most of the cases there was distinct pyrexia, and in the majority of cases the rash appeared to have a definite relation to the pyrexia, but in a few cases the rash preceded or succeeded the rise of temperature. Pains in the joints happened in some cases.

I have now given you many instances of "dermatitis" caused by more or less external agents, and others which operated more especially from within the system. We contemplated early in this address a marked irritation of the skin —those peculiar conditions known as "auto-graphism," or "dermo-graphism,"—and so on to undoubted instances of "dermatitis"; but before concluding my remarks, I wish to say a few words about a rare, but often very severe, and occasionally fatal, form of dermatitis, known as "pityriasis rubra," or by a still more expressive definition as "dermatitis exfoliativa." When fatal it is generally in consequence of the sequelæ which are associated with it. It is certainly one of the most extensive diseases of the skin known, and the exfoliative, rather than the bran-like shedding of the epiderm, is more typical of a tree shedding its leaves, as the scarf-skin comes away in large flakes. There the parity of the analogy must cease, because when once a tree has shed its leaves there are no more formed until another season, whereas the skin is so active in its reproduction of epidermis, and this activity may extend from the crown of the head to the sole of the foot, that there seems no end to the process, every part, even sometimes the hair and nails, being involved. So great desquamation is there that in the course of twenty-four hours a patient thus afflicted may deposit on his bed, or amongst his clothes, and on the floor of his room, as much as three or four pints of scales. A French writer says, "The patient is engaged in the labour of stripping himself involuntarily of his epidermis." At the commencement of the disease the skin becomes very hyperæmic, hence the term "rubra." This complaint may arise as early as the second year of life, and it has been met with at eighty-four years of age; but its most marked incidence is between forty and fifty years, and more males than females are affected by it.

In severe cases—for it has its phases—the hair may fall off and the nails may be thickened, and they too may be shed. When fatal—and the percentage of deaths is rather large—the fatality is generally caused by pneumonia (septic perhaps nearly always), bronchitis, or albuminuria, one or all, or even gangrene of parts may result. However, I must not go into a full description of this very interesting disease, but I have introduced the subject because I want to elucidate, from my point of view, an important factor in this disease, and in several others where there can be no want of unanimity of opinion.

We have spoken of peculiarities—idiosyncrasies—so that, comparatively speaking, only a few persons may be affected by certain external irritants or things taken internally, and we speak of pre-cancerous stages of cancer and pre-fungoid stages of that rare skin affection, mycosis fungoides, and some other conditions, in which the skin seems to pass through certain preparatory or preliminary phases, and in this way a "nidus" is made for various micro-organisms to develop in—nay, sometimes to revel in. Well, my own idea is that pityriasis rubra or dermatitis exfoliativa never begins absolutely as a primary disease. I have seen many cases—I use the word "many" advisedly—of this comparatively rare disease, but in every instance there has been a preliminary something before one could say the incidence of the complaint had commenced. It is but fair to state that several writers give it a "primary" standard occasionally.

We spoke a little while ago of certain changes that we noticed in the skin—physiological rather than strictly pathological – in that evanescent condition, dermo-graphia. Let us now go a few steps further. What happens in eczema erythematosum? Why should we get the peculiar reddened surface, the thickening and general puffiness of the skin, with the accentuated marks, that are so characteristic of this condition? I will now quote from a most interesting address given last May at St. John's Hospital, London, for Diseases of the Skin, by Prof. G. Sims Woodhead, of Cambridge: "On histological examination we find first of all that owing to the atrophy of the stratum granulosum and the filling of the spaces between or beneath the epithelium with fluid, the colour of the massed blood in the distended vessels of the corium shines through very distinctly, and we have the peculiar redness accounted for. We have, further, evidence of inflammation in the presence of escaped leucocytes accumulated in considerable numbers around these distended vessels, and of a more chronic inflammatory condition in the proliferation of the fixed connective tissue cells, some of which are rounded and embryonic, others of the more regular connective-tissue type. We have evidence of changes in the walls of the vessels, and perhaps also in the walls of the lymphatics, and of an impaired activity of the endothelium of these structures in the œdematous condition which assists very materially in causing a swelling of the parts; all this formation of new tissues and accumulation of fluids must necessarily lead to thickening of the skin, and we have at once an explanation of the naked-eye appearances. Moreover, we are led to look for (and to remove) some *irritant substance or condition* (the emphasis is mine) which may give rise to these histological and pathological changes so characteristic of a progressive inflammation set up by an irritant usually of an organised nature." Let us take another example. I am still quoting Prof. Woodhead: "In certain forms of eczema we see the results of the accumulation of the horny layers filled with coagulated albumin in which staphylococci can multiply and give rise to their products which, absorbed into the cutis, set up a series of changes well recognised by the expert." Unquestionably, bacteria and some of the higher fungi exert great influence in the production of skin diseases. "Furuncles, carbuncles, ringworm of various types, may all be grouped under this heading, whilst the acute inflammations of the skin, especially, in all probability, where vesicles are present, come, as regards causation, into this group. We have still to fathom the cause of varicella and variola, but these are probably due to the action of spore-bearing organisms which, making their way from the blood and lymphatic vessels to the lymph spaces of the skin, set up there the changes characteristic of these special diseases."

Purpura, tuberculosis and cancer of the skin, erysipelas, elephantiasis, and some other affections doubtless come into this class. "We find that this inflammation of the true skin is an essential part of most skin diseases, and it is interesting to note that it may be induced by irritant matter introduced either from the free surface or by the lymph and blood vessels from within, and all who have been occupied in the treatment of skin diseases are invariably impressed by the interdigitation of these two factors in their production." Do not these conditions assist us towards the explanation of many of the instances of dermatitis we have been reviewing? and is it to be wondered at that errors as to causation should sometimes arise?

And now, gentlemen, whilst my subject is by no means exhausted, yet I feel that I have trespassed upon your time and patience long enough. I have endeavoured to place some points upon a more intelligible basis, and if I have been successful in doing so, though in a small and humble way, I shall be very content, and I shall feel that I have done something—have placed a small stone upon our pathway of knowledge and a memorial tribute upon the monument of "our old and faithful friend."

Some years ago I spent several weeks in that very interesting and old city of Copenhagen, and chiefly with the object of studying the "Light Treatment" under the guidance of that great and able teacher, the late Professor Niels K. Finsen, and a very delightful and intellectual time I had; and during my stay the new Light Institution was opened. The objects it had in view were great, but their new University took a still higher flight. Over the gate of the principal entrance are a crest and motto. The crest is an eagle with outstretched wings, and its head turned up to heaven. The motto is this: "*Cœlestem adspicit lucem.*"

The Psalmist said ages ago: "For with Thee is the well of life, and in Thy light shall we see light." It is for increased light we are ever yearning and straining our eyes, and for this light our departed friend was one of the most eager searchers.

